# Ischemic Stroke Causes Disruptions in the Carnitine Shuttle System

**DOI:** 10.3390/metabo13020278

**Published:** 2023-02-14

**Authors:** Leonidas Mavroudakis, Ingela Lanekoff

**Affiliations:** Department of Chemistry—BMC, Uppsala University, 75237 Uppsala, Sweden

**Keywords:** acylcarnitines, mass spectrometry imaging, ischemic stroke, nano-DESI

## Abstract

Gaining a deep understanding of the molecular mechanisms underlying ischemic stroke is necessary to develop treatment alternatives. Ischemic stroke is known to cause a cellular energy imbalance when glucose supply is deprived, enhancing the role for energy production via β-oxidation where acylcarnitines are essential for the transportation of fatty acids into the mitochondria. Although traditional bulk analysis methods enable sensitive detection of acylcarnitines, they do not provide information on their abundances in various tissue regions. However, with quantitative mass spectrometry imaging the detected concentrations and spatial distributions of endogenous molecules can be readily obtained in an unbiased way. Here, we use pneumatically assisted nanospray desorption electrospray ionization mass spectrometry imaging (PA nano-DESI MSI) doped with internal standards to study the distributions of acylcarnitines in mouse brain affected by stroke. The internal standards enable quantitative imaging and annotation of endogenous acylcarnitines is achieved by studying fragmentation patterns. We report a significant accumulation of long-chain acylcarnitines due to ischemia in brain tissue of the middle cerebral artery occlusion (MCAO) stroke model. Further, we estimate activities of carnitine transporting enzymes and demonstrate disruptions in the carnitine shuttle system that affects the β-oxidation in the mitochondria. Our results show the importance for quantitative monitoring of metabolite distributions in distinct tissue regions to understand cell compensation mechanisms involved in handling damage caused by stroke.

## 1. Introduction

Ischemic stroke is one of the major causes of death worldwide and accounts for more than 80% of all stroke incidents [1]. To study ischemic stroke, middle cerebral artery occlusion (MCAO) is the most commonly used mouse model and it involves physical obstruction of blood flow towards part of the brain using an intraluminal suture [2]. The obstruction of blood flow that is the signature of stroke has detrimental effects on the homeostasis of cells and results in inflammation, oxidative stress, ionic imbalance, excitotoxicity, and finally apoptosis [3]. The oxidative stress induced due to the release of high intracellular levels of Ca^2+^, Na^+^ and adenosine diphosphate (ADP) is deleterious for the mitochondria. Since mitochondria are the cells’ primary energy production stations this is largely affected by ischemic stroke.

The mitochondria can produce energy through the oxidation of fatty acids (FAs), which is an alternative when the essential nutrients, such as glucose, are not available to the brain cells. However, β-oxidation, the process of oxidizing FAs to produce acetyl-CoA, is not as favorable as glucose oxidation because it requires 15% more oxygen, produces superoxides, and generates less ATP [4]. Nevertheless, up to 20% of energy requirements can be provided by FA oxidation in brain [5].

Oxidation of long-chain (C14-C20) FAs is a multistep process that requires their transport into the mitochondria. Specifically, they are imported through the carnitine shuttle system while the shorter-chain (C3-C12) FAs simply diffuse through the mitochondrial membrane [6,7]. Once the FAs are activated and in the form of acyl-CoA esters, they are coupled with carnitine through the enzyme carnitine palmitoyltransferase 1 (CPT1) and transported through the outer mitochondrial membrane. Following, the acylcarnitines (ACs) are transported across the inner mitochondrial membrane using the carnitine-acylcarnitine translocate (CACT). Finally, carnitine palmitoyltransferase 2 (CPT2) catalyzes the decoupling of the carnitine moiety from the acylcarnitines in order to produce fatty acyl-CoA that can enter the cycle of β-oxidation for energy generation. By use of individual acylcarnitines concentration, or their ratios to carnitine, several studies have assessed the activity of CPT1 and CPT2 in biological systems [8,9,10,11,12].

The energy status of biological systems monitored through analysis of carnitine and ACs is typically performed by coupling liquid chromatography (LC) to mass spectrometry (MS) [12,13,14]. Although such approaches are highly robust and sensitive, the necessary sample homogenization prohibits the determination of analyte distribution within selected parts or regions of the tissue. An attractive alternative is therefore to use mass spectrometry imaging (MSI) techniques, which can provide localized information on ACs and thereby directly assess the cells’ energy status in intact tissue sections [15,16,17]. Nanospray desorption electrospray ionization (nano-DESI) is an MSI technique that uses a localized liquid extraction of analytes from the tissue surface. In short, the analytes are desorbed from the tissue into a liquid bridge flowing between two fused silica capillaries positioned in front of the mass spectrometer [18,19]. Following, the desorbed analytes are transported through the second fused capillary towards the inlet of the mass spectrometer and ionized by electrospray or pneumatically assisted (PA) electrospray due to vacuum inside the MS or the Venturi effect, respectively [20]. By moving the sample under the liquid bridge, data are continuously acquired for subsequent construction of 2-D maps showing analyte distributions in the tissue. Each pixel on the constructed 2-D maps corresponds to the intensity of a selected ion from each scan event through the data acquisition. In nano-DESI, quantitation is enabled by addition of standards to the nano-DESI solvent and challenging analytes can be targeted by addition of reagents for reactive chemistry [21,22,23,24].

Here, we have employed PA nano-DESI MSI to study the energy status of the damaged cellular region in ischemic stroke by mapping the distribution of ACs in the MCAO stroke model. The distributions and annotations of endogenous ACs were confirmed using deuterated standards and fragmentation patterns. Finally, we report the increased activity of CPT1 and CPT2 in the brain hemisphere damaged by ischemic stroke compared to the healthy hemisphere. Overall, our results suggest impaired transportation of FAs through the carnitine shuttle system due to ischemia. 

## 2. Materials and Methods

### 2.1. Chemicals and Materials

Methanol and formic acid (98–100%) were purchased from Merck (LC-MS grade). Deionized water (18.2 MΩ) was obtained from an in-house MilliPore purification system. L-carnitine-HCl-(methyl-d3) (referred to herein as C-d3) and octadecanoyl (18,18,18–d3)-L-carnitine-HCl (referred to as C18-AC-d3) were purchased from Larodan AB (Solna, Sweden). D-Glucose-6,6-d2 (referred to as glucose-d2), phosphatidylcholine 11:0/11:0 (PC 22:0) and oleic acid-d9 were purchased from Merck. 

### 2.2. Pneumatically Assisted (PA) Nano-DESI

The PA nano-DESI probe was constructed using fused silica capillaries (50 µm ID and 150 µm OD Polymicro Technologies LLC, Pheonix, AZ, USA) fixed in an in-house developed 3D printed casing that kept the primary and the secondary capillary in position [20]. A backpressure of 2.5 bar nitrogen gas was used for pneumatically assisted nanospray. The solvent was methanol:water 9:1 *v*/*v* and 0.1% formic acid spiked with known concentrations of internal standards and was delivered through the primary capillary using a syringe pump (Legato 180, KD Scientific, Holliston, MA, USA) [22,25]. Dataset A was acquired at a flowrate of 0.5 µL min^−1^ with the solvent containing 0.2 µM c-d3, 0.2 µM C18-AC-d3, 30.5 µM glucose-d2 and 4 µM PC 22:0. Dataset B was acquired at a flowrate of 0.3 µL min^−1^ with the solvent containing 0.4 µM C-d3, 0.1 µM C18-AC-d3, 30.5 µM glucose-d2, 2 µM oleic acid-d9, and 4 µM PC 22:0.

### 2.3. Middle Cerebral Artery Occlusion Mouse Model

All animal experiments were performed by Creative Biolabs Inc. (Shirley, NY, USA) and MCAO brains from mice were purchased and sectioned in our facilities. Specifically, MCAO was induced in 8–10 weeks old male C57BL/6 mice for 1 h followed by 2 h of reperfusion prior to sacrificing the animals and removing the intact brain. Three flash frozen MCAO mouse brains were purchased and sectioned coronally using a Leica CM1900 cryotome (Leica Microsystems) at 10 µm thickness. The tissue sections were thaw-mounted on regular glass slides and stored at −80 °C until analysis. In total, 17 tissue sections from three animals were analyzed, 8 in dataset A and 9 in dataset B.

### 2.4. Mass Spectrometry Imaging

The glass slide with the tissue section was mounted on an X-Y-Z motorized stage (Zaber Technologies Inc., Vancouver, BC, Canada) that was controlled by a custom designed LabVIEW program [26]. The sample was moved at a rate of 0.04 mm s^−1^ along the x-axis direction with a step along the y-axis of either 150 µm or 75 µm for improved spatial resolution through oversampling [27]. The calculated pixel size along the x-axis was ~24 µm based on the scan rate of the mass spectrometer (1.7 scans s^−1^) and the stage scanning speed (40 µm s^−1^). Along the y-axis, the pixel size is determined by the stepping size of the stage. The data were recorded using a QExactive mass spectrometer (Thermo Fischer Scientific, Bremen, Germany) in the positive ion mode between *m/z* 70–1000 with an electrospray voltage of 3.5 kV applied directly on the syringe delivering the solvent. The mass spectrometer was operated at resolution of 140,000, ion transfer capillary temperature of 300 °C, automatic gain control (AGC) target of 1e6, maximum injection time of 300 ms with a typical injection time (IT) of 2 ms, and S-lens RF level of 50. 

### 2.5. MS/MS of Carnitine Standards and Endogenous Carnitines

For identification of endogenous carnitines in the tissue, a Parallel Reaction Monitoring (PRM) method was set up in the positive ion mode with an inclusion list comprised of various putatively identified ACs and added standards (Table S1). The AGC target of each MS/MS event was 2e5 with a maximum inject time of 300 ms and an isolation window of 0.4 *m/z*. Higher-energy collisional dissociation (HCD) was applied in a stepped manner (at normalized collision energies (nCE) from 10 to 30). For the MS/MS experiments with PA nano-DESI, the conditions were the same as for acquisition of dataset B. The probe was positioned on an ischemic area of an MCAO mouse brain tissue section and slightly moved in the area to continuously desorb endogenous molecules.

### 2.6. Data Processing and Statistical Analysis

After data collection, Thermo RAW files were converted to mzML files using MSConvert (Proteowizard) [28]. The mzML files were processed using MATLAB R2022a (MathWorks, USA) with in-house developed scripts for obtaining the intensity of target *m/z* values from each data file within 5 ppm of mass error tolerance. For ion image normalization, the intensity of the endogenous ion in each pixel was divided by the intensity of the appropriate internal standard. Region of interest (ROI) of areas corresponding to the healthy and the ischemic part of the tissue were selected using the in-house script for further interrogation. The ischemic ROI was selected using ions known to accumulate [23,29,30], verified by overlaying with the optical image, and the healthy ROI was selected as mirror with a similar amount of pixels (Table S2). The average intensities of selected ions were obtained from each ROI and statistical analysis was conducted using a two-tailed Wilcoxon rank sum test. For statistical analysis, data within the 5^th^ and 95^th^ percentile of the dataset were considered. 

## 3. Results

### 3.1. Matrix Effects between Healthy and Ischemic Region Are of Equal Magnitude

Matrix effects in MS are inherently challenging, especially in MSI where a pre-separation step is omitted. In principal, matrix effects during ionization result in ion suppression or ion enhancement and two types can be observed; alkali metal ion related and molecular composition related [21]. Imaging with nano-DESI enables internal standards to be included in the nano-DESI solvent for quantitation and to study matrix effects, such as ion suppression. The magnitude of ionization suppression during imaging was studied using the two standards C18-AC-d3 and C-d3. Following, the amount of suppression was calculated using the absolute difference between the average intensity of one standard in a tissue area (I_tissue_ in Equation (1)) and the average intensity of the same standard on an area on glass (I_glass_ in Equation (1)), divided by its intensity on a glass area (Equation (1)).

% Suppression = (|I_tissue_ − I_glass_|)/I_glass_ × 100 (1)

**Figure 1:**
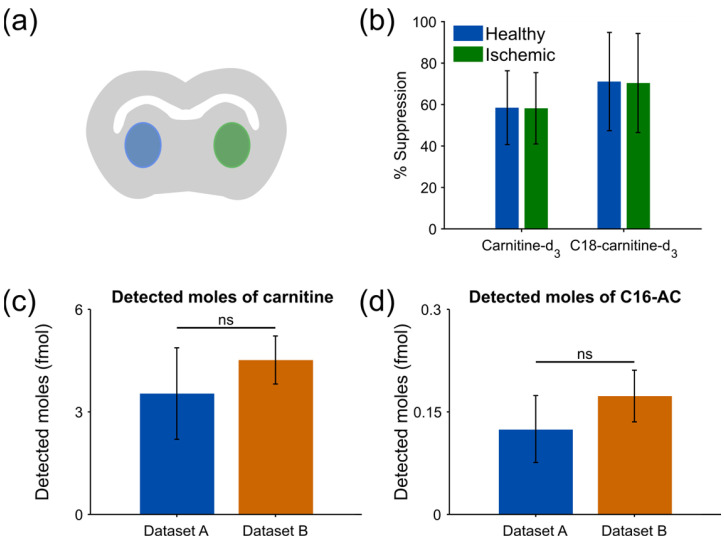
Comparison of ion suppression and quantitative assessments. (**a**) Schematic of a mouse brain tissue section where the healthy area is depicted as blue and the ischemic as green. (**b**) Ionization suppression (%) of C-d3 and C18-AC-d3 between healthy and ischemic tissue regions. (**c**) Detected moles of carnitine in the healthy region from dataset A and B using Equation (2). (**d**) Detected moles of C16-AC in the healthy region from dataset A and B using Equation (2). The error bars in (**b**) represent the propagated error due to the standard deviation of each scan in Equation (1).

The average tissue intensities were obtained from the areas shown in Figure 1a, where blue corresponds to the healthy region and green to the region damaged by ischemic stroke (Table S3). From Equation (1), it follows that an analyte with zero intensity on the tissue area would have 100% suppression and that an analyte that is not affected by ion suppression would have 0% ion suppression and thereby a conserved intensity on the tissue compared to the glass. The results show a slightly overall higher suppression of ~12% for the C18-AC-d3 compared to C-d3 on the tissue in both the healthy and ischemic areas (Table S4). However, importantly, neither standard shows a difference in ionization suppression between the two regions (Figure 1b).

### 3.2. Use of Internal Standards Allows for Robust Relative Quantification of Endogenous Molecules

The inclusion of appropriate internal standards in the PA nano-DESI solvent also allows for relative quantitation of detected endogenous analytes. This is typically achieved using a one-point calibration where the intensity ratio of the analyte to the internal standard (I_end_/I_IS_) is multiplying with the concentration of the internal standard (C_IS_, µM) [31] (Figure S1). Furthermore, the detected moles (n_end_) in each mass spectrum can be calculated by including the solvent flowrate (F, µL min^−1^) and the scan time between two subsequent scan events (ST, milliseconds) (Equation (2)).

n_end_ = (I_end_/I_IS_) × C_IS_ × F × ST, (2)

Here, we demonstrate that by accounting for the solvent flow rate, the quantitative approach can accommodate datasets acquired with different flow rates and concentrations of the internal standards. In particular, we show that there is no significant difference between the detected amounts of endogenous carnitine and C16-AC for datasets A and B despite the different flow rates and concentrations of internal standards during acquisition (Figure 1c,d). Overall, this is of importance when comparing datasets acquired under slightly different conditions.

### 3.3. Identification of Endogenous Acylcarnitines through MS/MS

**Figure 2:**
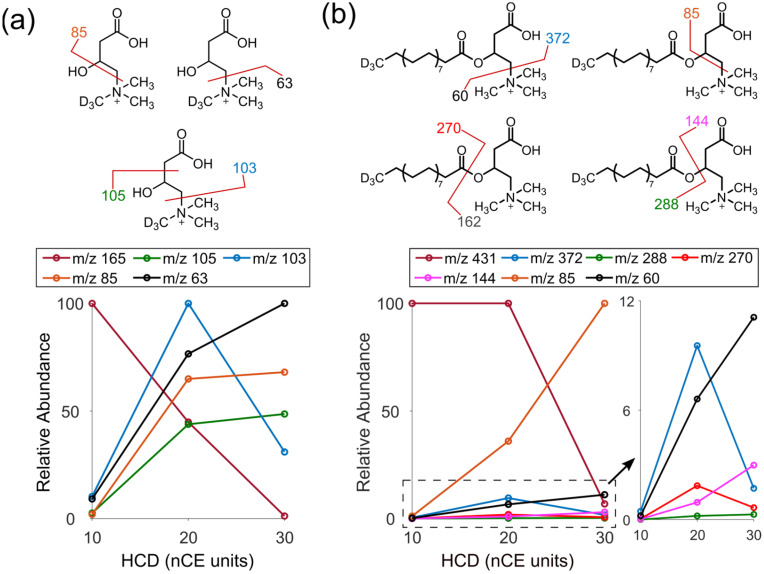
Fragmentation studies of C18-AC-d3 and C-d3 by MS/MS and varied collision energy. Proposed fragmentation sites and relative abundance of product ions for (**a**) C-d3 and (**b**) C18-AC-d3. The fragments of *m/z* 63 and 60 both correspond to the trimethylamine group but with and without three deuterium atoms, respectively.

Annotation of analytes with mass spectrometry imaging mainly relate to product ion formation in MS/MS experiments, therefore, the fragmentation patterns of the protonated standards C-d3 and C18-AC-d3 were studied. Both standards show the main fragmentation sites around the head group [14,32], despite their differences in size. Furthermore, product ions associated with the C18-AC-d3 acyl chain are only detected at low abundances (Figure 2a,b, Figure S2 and Figure S3). However, when investigating different HCD levels, it is clear that the C-d3 and C18-AC-d3 have different fragmentation efficiency of the respective sites. 

At HCD nCE of 20 units, C-d3 predominantly loses the trimethylamine group and leaves a base peak product ion at *m/z* 103 (Figure 2a and Figure S2). For C18-AC-d3, the dominating product ion is instead formed from the loss of both the trimethylamine group and the acyl chain, which leaves the product ion at *m/z* 85 (Figure 2b and Figure S3). Additional product ions of C18-AC-d3 are in much less abundance, including the product ion at *m/z* 372, which is produced through the same fragmentation pathway as the *m/z* 103 of C-d3.

At nCE of 30, the distribution of product ions for C-d3 is shifted and the deuterated trimethylamine group at *m/z* 63 becomes most abundant while the *m/z* 103 has the lowest relative abundance among the product ions. For C18-AC-d3, the dominating product ion of is still the *m/z* 85. However, at much lower abundances the resemblance to the fragmentation pattern of C-d3 is high with an increased abundance of the trimethylamine group at *m/z* 60 (Figure 2b insert). It seems that the presence of the acyl chain in C18-AC-d3 induces a more efficient fragmentation towards the product ion *m/z* 85. Overall, compared to C-d3, the larger ACs appear to fragment less efficiently at lower HCD (20 nCE), which indicates a structure-related stability during the activation step of the HCD process. Importantly, these differences in fragmentation efficiency are crucial for annotating ACs based on MS/MS and for obtaining the highest sensitivity in selected reaction monitoring (SRM) or multiple reaction monitoring (MRM).

Based on our findings, thirteen endogenous ACs were annotated directly from tissue using a PRM method with stepped HCD. The precursors of the inclusion list, the detected *m/z* value, the characteristic fragments, and the assignments of carnitines detected directly from the tissue section are summarized in Table S1.

### 3.4. PA Nano-DESI MSI of MCAO Stroke Model

**Figure 3:**
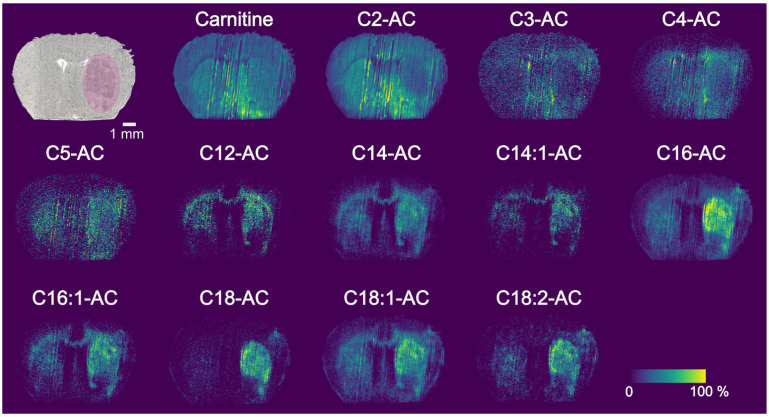
Ion images of various carnitines obtained through PA nano-DESI MSI. The optical image of MCAO mouse brain tissue section shows the pink area as the ischemic region. The ion images of carnitine and various acylcarnitines are depicted as normalized intensities to carnitine-d3 (C to C5) or C18-carnitine-d3 (C12 to C18). Color map scale shows relative intensity.

Imaging with PA nano-DESI MSI readily provides ion images of carnitine and ACs with various chain lengths. In addition to the thirteen identified endogenous acylcarnitines, the tissue could include ACs with –OH modifications or acyl chain between 6 and 11, and over 18. (Figure 3 and Table S1). Generally, the detected intensities of C3-, C4-, C5-, C12- and C14:1-AC were lower compared to the other ACs. All ion images in Figure 3 are normalized to its closest related internal standard, the small endogenous C-C5 to the C-d3 and the larger C12-C18 to the C18-AC-d3, although no difference in distribution can be observed depending on normalization (Figure S5 and Figure S6). Two different trends of distributions in brain tissue are observed (Figure 3 and Figure S4). Specifically, carnitine and C2-AC localize similarly throughout the brain section with higher localization in the regions of hypothalamus (HY) and thalamus (TH). The rest of the detected ACs are found mainly in white matter regions (corpus callosum, cc) and in caudoputamen (CP).

The ischemic area of the tissue is located in the right hemisphere of the brain and specifically in the caudoputamen (CP) (Figure 3, Figure S4 and Figure S5). This was confirmed both by the decreased abundance of K^+^/Na^+^ adduct ratios of standard PC 22:0 as well as the optical image (Figure S7) [21,23,33]. Upon visual inspection, long-chain ACs such as C14-, C14:1-, C16-, C16:1, C18-, C18:1-, C18:2- have a higher abundance in the ischemic region, with the most notable difference in the ion image of C18-carnitine. However, to fully realize the metabolite differences inflicted by ischemia, the average detected amounts need to be assessed from a larger dataset through ROI analysis.

### 3.5. Region of Interest Analysis

**Figure 4:**
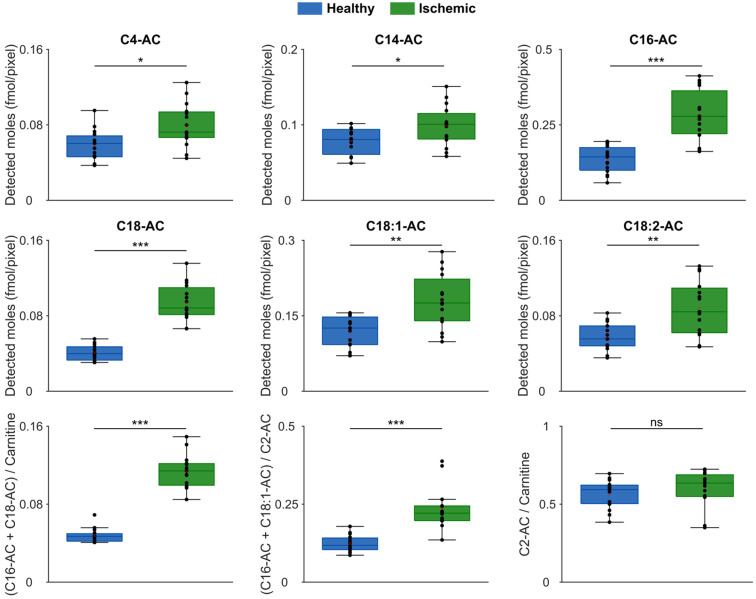
Accumulation of ACs and enzymatic activity. ROI analysis of significantly altered ACs that accumulated in the ischemic region (top two rows) and ratios of (C16-AC + C18-AC)/carnitine depicting CPT1 activity, (C16-AC + C18:1-AC)/C2-AC depicting CPT2 activity and C2-AC/carnitine depicting β-oxidation activity (bottom row). Statistical significance was assessed using a two-tailed Wilcoxon rank sum test where significant differences (* *p* < 0.05, ** *p* < 0.01, *** *p* < 0.001) are denoted with asterisk(s).

To enable statistical comparison of the localization of metabolites in the tissue, ROI analysis was performed by selecting the ischemic and the healthy regions and averaging the intensities of the metabolites of interest. Following, the raw average intensities of each endogenous molecule were converted to detected moles per pixel using the respective internal standard and Equation (2). Statistical comparisons of 16 tissues sections were performed using a Wilcoxon rank sum test (two-tailed) and one section was removed as an outlier. The results show that C4-, C14-, C16-, C18-, C18:1-, C18:2-AC and LPC 16:0 are accumulating 1.26- to 2.3-fold in the ischemic region (Figure 4, Figure S8 and Table S5). Additionally, carnitine, C2-, C3-, C5-,C12-AC, and free FAs 18:1 and 18:2 were found at similar amounts in the healthy and ischemic regions (Figure S8 and Figure S9). Finally, glucose was found to be significantly decreased in the ischemic region (Figure S8).

Since ACs are actively transported into the mitochondria for subsequent β-oxidation (Figure S10), the enzymatic activity of CPT1 and CPT2 was calculated based on the concentration of individual ACs [6,9,34]. Specifically, the ratio of (C16-AC + C18-AC)/carnitine was used for CPT1 and (C16-AC + C18:1-AC)/C2-AC for CPT2. It was found that the activity of both CPT1 and CPT2 was elevated in the ischemic region (Figure 4). Furthermore, it was found that β-oxidation, determined using the ratio of C2-AC/carnitine, was conserved despite ischemia (Figure 4).

## 4. Discussion

The onset of ischemic stroke keeps essential nutrients such as oxygen and glucose from reaching the ischemic region, which causes cell damage and forces cells to use alternative strategies for energy [35]. The use of internal standards and annotation with MS/MS in combination with MSI enables the simultaneous assessment of ischemic stroke on thirteen detected AC species. In energy generation through β-oxidation, long-chain ACs (C12-C20) are important for introducing activated long-chain FAs into the mitochondria (Figure S10) [6,34]. The detected accumulation of C14-, C16-, C18-, C18:1- and C18:2-AC in this study is a well-known indicator of disrupted transportation and oxidation of FAs [12,13,34]. This suggests that ischemic stroke causes metabolic dysfunctions in the carnitine shuttle system that limit the use of long-chain FAs for energy production and ultimately cell survival [12]. Additionally, accumulation of C4-AC in the ischemic area provides evidence that short-chain FA metabolism is also disturbed [36,37]. In particular, the accumulation of the short-chain C4-carnitine in hypoxic-ischemic encephalopathy has been previously linked to mitochondrial failure and reported as a result of inhibition or defects of short-chain acyl-CoA dehydrogenase [36,37].

By assessing enzymatic activity using the ratio of acylcarnitines to free carnitine or acetylcarnitine [6,9,34], our data provide evidence for increased activity of CPT1 and CPT2 in the ischemic region of the tissue and no change in β-oxidation (Figure 4). This clearly demonstrates that the carnitine shuttle system is disrupted; although the point of disruption cannot be distinguished. Generally, it would be reasonable to assume that increased amounts of long-chain acylcarnitines is a consequence of increased CPT1 action and a decreased CPT2 activity [38,39,40]. However, this is not supported by our data (Figure 4). Nevertheless, it cannot be ruled out that the net effect we observe is due to an increased CPT2 activity on top of an even higher activity of CPT1. CPT1 is the rate-limiting enzyme in the carnitine shuttle system and its increased activity could be justified by the lack of its natural inhibitor; malonyl-CoA [41,42,43]. Malonyl-CoA is produced mainly via the metabolism of glucose but with the evidenced decreased amount of glucose in the ischemic area, it can be expected that the levels of malonyl-CoA are also reduced (Figure S8) [41,44]. Consequently, inhibition of CPT1 would be limited and result in the detected accumulation of long-chain acylcarnitines. Furthermore, an increased activity of CPT1 can lead to excess conversion of the long-chain FAs 18:1 and 18:2 to the corresponding acylcarnitines, which would explain the lack of FA 18:1 and 18:2 accumulation in our data (Figure S8) [13]. It would be of interest in future work to monitor the enzymatic activities in the carnitine-shuttle system during ischemia to further elucidate the main contributing step to the disruption and elucidate contributions from the previously reported isoforms [41]. Gaining a deeper understanding of the molecular mechanisms activated during stroke will greatly contribute to future targeting of enzymes for treatment and damage alleviation.

## 5. Conclusions

It is well known that stroke is a detrimental condition for cell survival. By imaging with PA nano-DESI MSI we show that C4-AC and long-chain ACs accumulate in the ischemic region of the brain after MCAO. By quantifying thirteen carnitine species, we estimate the enzymatic activity of the carnitine transporting enzymes CPT1 and CPT2 by calculating carnitine ratios. Despite our finding that both CPT1 and CPT2 are increased in the ischemic region, we hypothesize that the activity of CPT1 is more elevated due to the lack of its natural inhibitor malonyl-CoA that is usually formed through commonly used energy generating pathways. Overall, our findings are consistent with prevailing theories using bulk analysis and show for the first time the additional dimension of the distinct distributions and abundances of long-chain ACs in ischemic stroke brain tissue.

## Data Availability

The raw data presented in this study are available on request from the corresponding author. The data are not publicly available due to privacy.

## Supplementary Materials

The following supporting information can be downloaded at: https://www.mdpi.com/article/10.3390/metabo13020278/s1, Table S1: MS/MS data of endogenous carnitines from MCAO brain; Table S2: Number of ROI pixels in healthy and ischemic regions; Table S3: Raw data for Figure 1b; Table S4: Ionization suppression data (%) from Figure 1b; Figure S1: One-point calibration data for carnitine and C16-carnitine; Figure S2: MS/MS spectra of carnitine-d3; Figure S3: MS/MS spectra of C18-carnitine-d3; Figure S4: Ion images of carnitine and C16-carnitine from three biological replicates; Figure S5: Ion images of carnitines with different normalizations; Figure S6: Image of carnitine-d3/C18-carnitine-d3 ratio; Figure S7: Image of PC 22:0 K^+^/Na^+^ adduct ratio; Table S5: Average fold change of accumulated acylcarnitines in the ischemic area; Figure S8: Ion images of glucose, LPC 16:0 and ROI analysis of glucose, LPC 16:0, FA 18:1, FA 18:2; Figure S9: ROI analysis of carnitine, C2-, C3-, C5- and C12-carnitine; Figure S10: Schematic overview of the carnitine shuttle system.
