# Supplementary material for: Ischemic Stroke Causes Disruptions in the Carnitine Shuttle System
Source: Metabolites. 2023 Feb 14;13(2):278. doi: 10.3390/metabo13020278 (PMC9968086; doi:10.3390/metabo13020278)
Supplement: Supplementary file 1 [file metabolites-13-00278-s001.zip › metabolites-2146264-supplementary.pdf]

Supplementary material

Ischemic stroke causes disruptions in the carnitine shuttle system

Leonidas Mavroudakis[1], and Ingela Lanekoff[1],*

[1]Department of Chemistry – BMC, Uppsala University, 75237 Uppsala, Sweden
*Correspondence: Ingela.lanekoff@kemi.uu.se

Table S1. MS/MS data of standards spiked in the PA nano-DESI solvent, endogenous carnitine and acylcarnitines from an ischemic mouse brain tissue section

Precursor m/z	Detected m/z[a]	Characteristic fragments	Assignment[b]
165.1314	165.1314	63.0998, 85.0287, 103.0394, 105.1106	L-carnitine-(methyl-d3)[c]
431.3920	431.3920	60.0810, 85.0287, 144.1022, 270.2877, 372.3193	octadecanoyl (18,18,18-D3)-L-carnitine[c]
162.1125	162.1125	60.0811, 85.0287, 103.0395	Carnitine
204.1230	204.1231	60.0811, 85.0287, 145.0498	C2-carnitine
218.1387	218.1390	60.0811, 85.0287, 144.1022	C3-carnitine
232.1543	232.1547	60.0810, 85.0287, 173.0810	C4-carnitine
246.1700	246.1703	60.0810, 85.0287, 187.0967	C5-carnitine
344.2795	344.2798	60.0810, 85.0287, 285.2068	C12-carnitine
372.3110	372.3109	60.0810, 85.0287, 211.2060, 313.2381	C14-carnitine
370.2952	370.2951	60.0810. 85.0287	C14:1-carnitine
400.3421	400.3420	60.0811, 85.0287, 144.1022, 239.2375, 341.2694	C16-carnitine
398.3265	398.3264	60.0811, 85.0287	C16:1-carnitine
428.3734	428.3729	60.0811, 85.0287, 144.1022, 267.2688, 309.2795, 369.3006	C18-carnitine
426.3578	426.3576	60.0811, 85.0287, 144.1022, 265.2532, 367.2852	C18:1-carnitine
424.3421	424.3420	85.0287, 60.0811, 144.1022, 263.2375	C18:2-carnitine

[a]Refers to the m/z detected during the MSI experiments.
[b]All assignments correspond to protonated adducts.
[c]Standard included in the nano-DESI solvent.

Table S2. Number of selected ROI pixels among the analyzed tissue sections

Sample	Ischemic area	Healthy area
aa9_2	3368	3290
aa9_3	2104	1550
aa9_4	1838	1382
bb4_3	1988	1772
bb4_4	2236	1880
bb5_1	2220	2377
cc2_2	1375	1196
cc2_3	1446	1213
cc2_4	1256	1147
aa6_1	2126	1850
aa6_4	1714	1520
bb3_2	1314	1229
bb3_3	4112	3554
bb3_4	533	530
cc1_2	854	808
cc1_4	854	808
cc1_5	1826	1329

Table S3. Raw average intensities of carnitine-d3 and C18-carnitine-d3 from the healthy, ischemic and glass area obtained from one ion image. The data were used for calculating the ionization suppression magnitude shown in Fig 1b.

Standard	Healthy area	Ischemic area	Glass area
Carnitine-d3	2.03E+06 ± 6.25E+05	2.05E+06 ± 5.85E+05	4.90E+06 ± 5.23E+05
C18-carnitine-d3	1.39E+06 ± 3.23E+05	1.42E+06 ± 3.61E+05	4.80E+06 ± 8.89E+05

Table S4. Ionization suppression (%) of carnitine-d3 and C18-carnitine-d3 among the healthy and ischemic region. The data are also presented in Fig. 1b and the error bars depict the propagated error due to the standard deviation of each intensity term in eq. 2 of main text.

Standard	Healthy area	Ischemic area
Carnitine-d3	58.5 ± 17.8	58.2 ± 17.2
C18-carnitine-d3	71.1 ± 23.7	70.4 ± 23.9

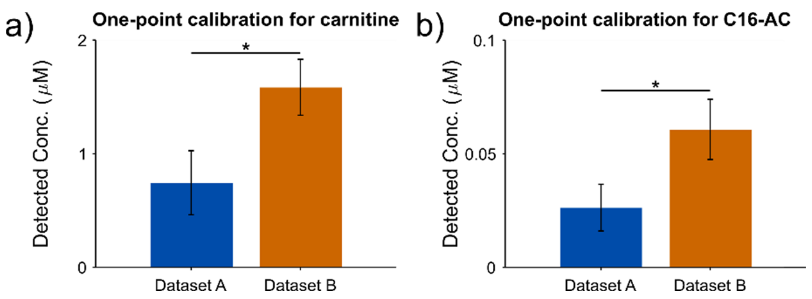

Figure S1. Detected concentrations of carnitine (a) and C16-carnitine (b) from two different datasets (see "Experimental Design" in SI) using one-point calibration relative quantitation. The means of the two datasets were statistically significant using a Wilcoxon rank sum test (*p<0.05).

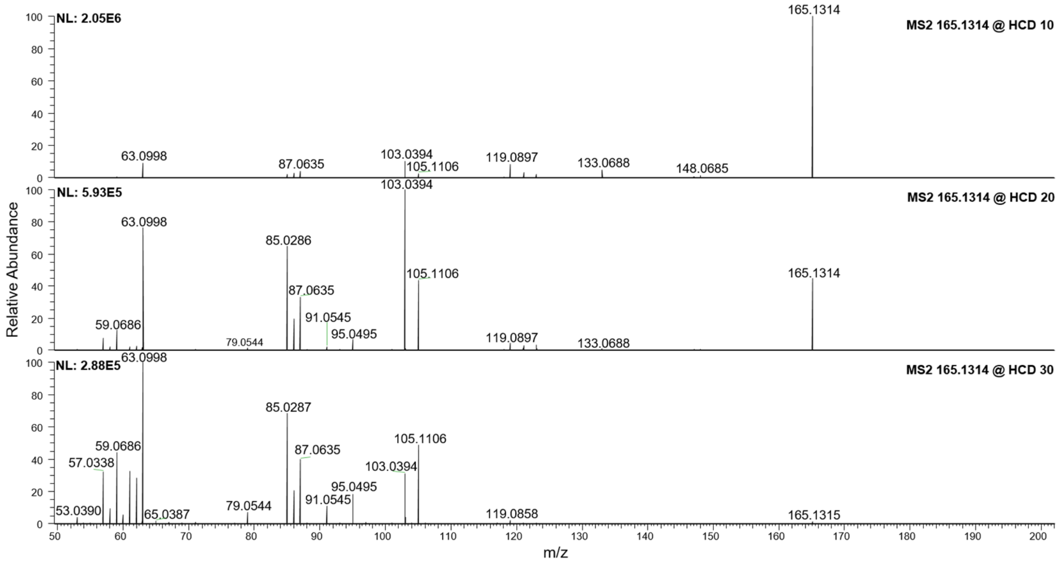

Figure S2. MS/MS spectra of carnitine-d3 (L-carnitine-(methyl-d3), m/z 165.1315) at various HCD levels.

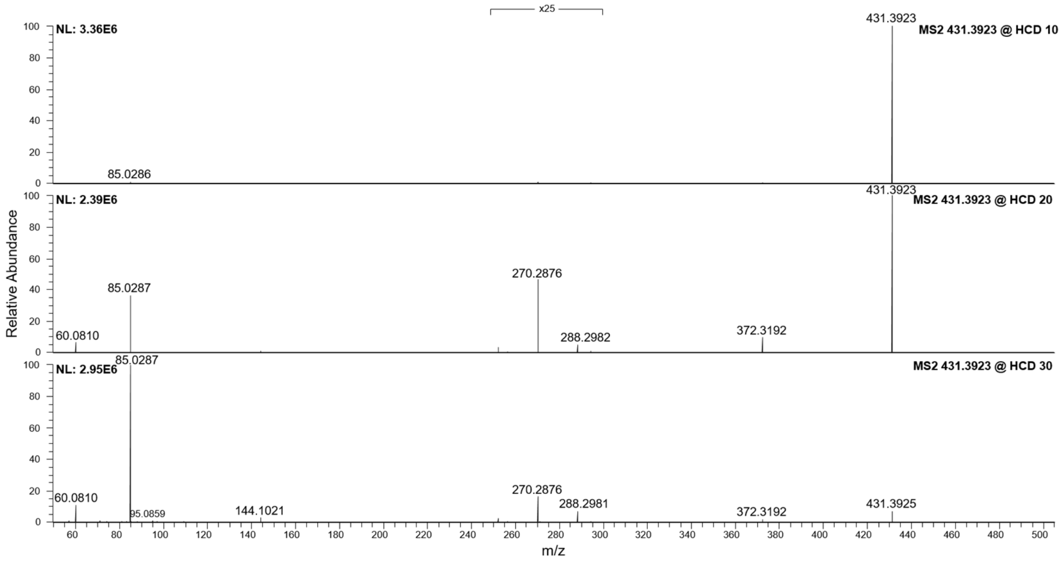

Figure S3. MS/MS spectra of C18-carnitine-d3 (octadecanoyl (18,18,18)-d3-L-carnitine, m/z 431.3925) at various HCD levels.

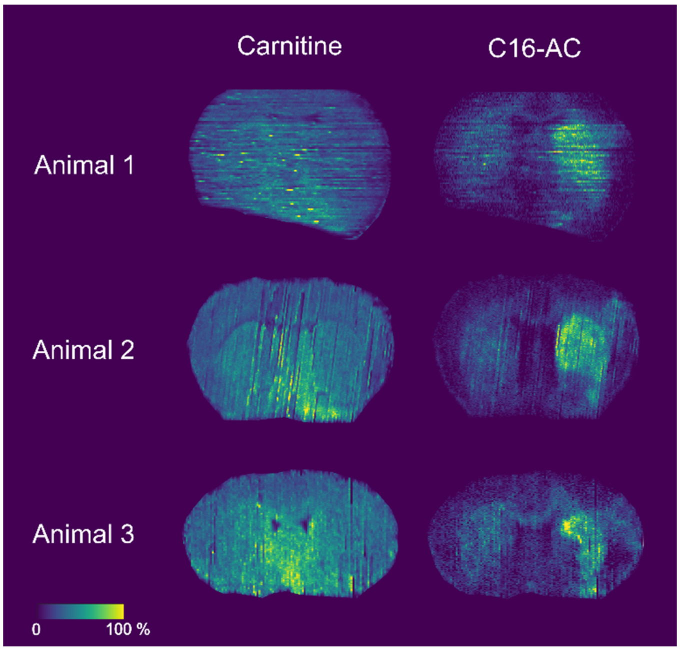

Figure S4. Molecular distributions of carnitine and C16-carnitine in three different tissue sections from biological replicates.

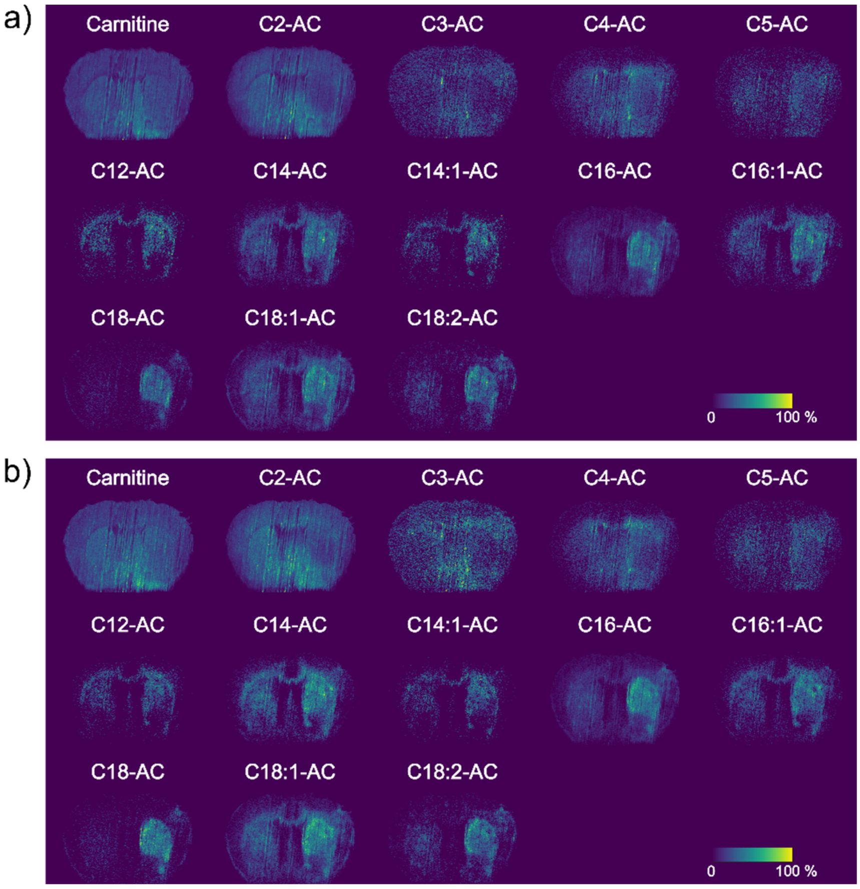

Figure S5. Ion images of endogenous carnitines normalized to carnitine-d3 (a) or C18-carnitine-d3 (b).

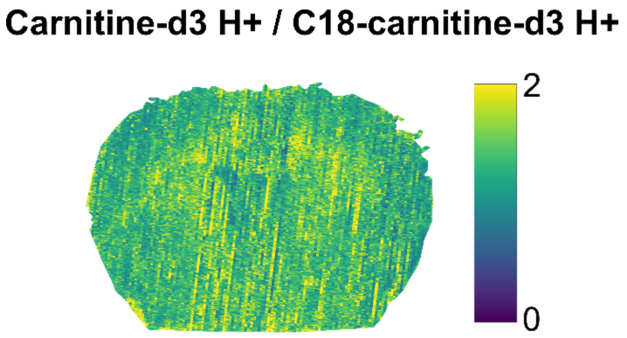

Figure S6. Image depicting the ratio of carnitine-d3 / c18-carnitine-d3 as protonated adducts.

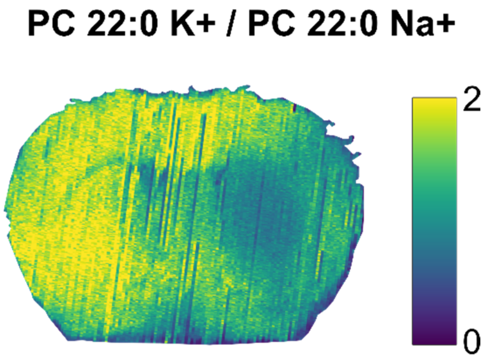

Figure S7. Image depicting the ratio of PC 22:0 K+ / PC 22:0 Na+. The ischemic area is identified by the decreased ratio of K^+/Na^+ adduct distribution on the right part.

Table S5. Average (± standard deviation) fold change of acylcarnitines that show accumulation in the ischemic area.

Acylcarnitine	Average fold change
C14-AC	1.26 ± 0.22
C16-AC	2.10 ± 0.13
C18-AC	2.31 ± 0.27
C18:1-AC	1.55 ± 0.12
C18:2-AC	1.55 ± 0.25

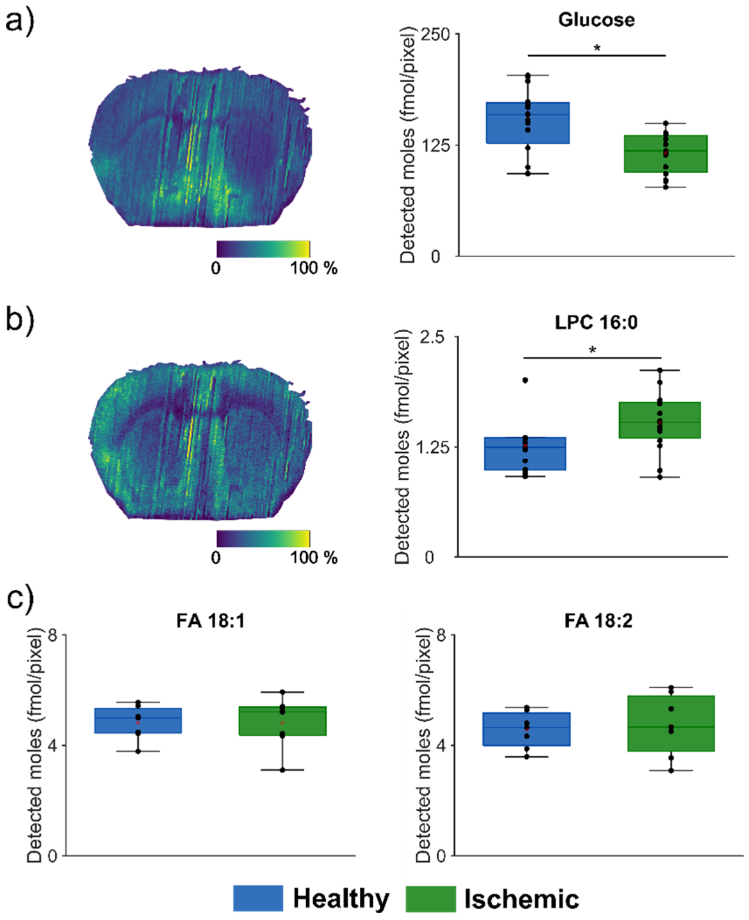

Figure S8. A) Ion image of glucose normalized to glucose-d2 depicted as sodiated adduct and ROI analysis. B) Ion image of lysophosphatidylcholine (LPC) 16:0 normalized to phosphatidylcholine (PC) 22:0, depicted as sodiated adduct and ROI analysis. C) ROI analysis of fatty acid (FA) 18:1 and 18:2 as sodiated adducts and normalized to oleic acid-d9.

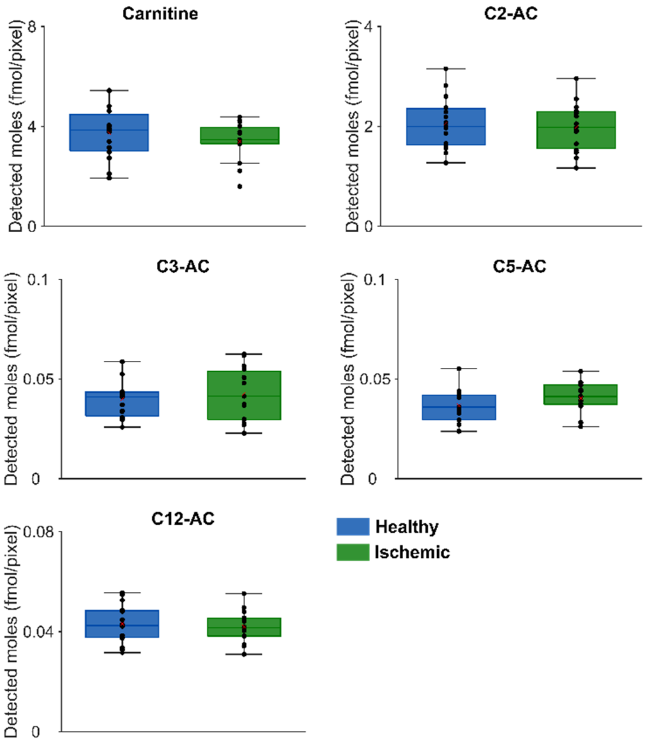

Figure S9. ROI analysis of ischemic and healthy region in MCAO mouse brain. Shown are the detected moles of carnitine, C2 carnitine, C3 carnitine, C5 carnitine and C12 carnitine. The two regions were not found to have statistically significant different means when using a two-tailed Wilcoxon rank sum test.

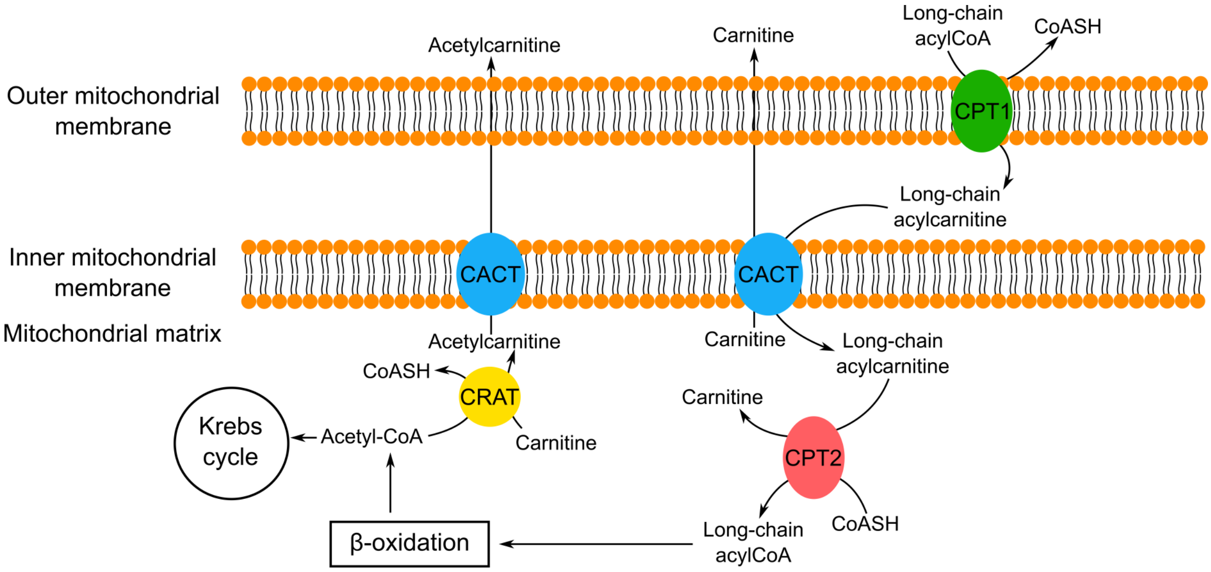

Figure S10. Schematic overview of the carnitine shuttle system. CPT1: carnitine palmitoyltransferase 1, CPT2: carnitine palmitoyltransferase 2, CACT: carnitine-acylcarnitine translocase, CRAT: carnitine acetyltransferase
